# Banded gastric bypass - four years follow up in a prospective multicenter analysis

**DOI:** 10.1186/1471-2482-14-88

**Published:** 2014-11-12

**Authors:** Luc Lemmens, W Konrad Karcz, Waleed Bukhari, Jodok Fink, Simon Kuesters

**Affiliations:** Antwerp Medical Center, Antwerp, 2000 Belgium; Department of Surgery, University of Schleswig-Holstein, Campus Lübeck, Ratzeburger Allee 160, 23538 Lübeck, Germany; International Medical Centre, Jeddah, Kingdom of Saudi Arabia; Department of General and Visceral Surgery, University of Freiburg, Freiburg, Germany

**Keywords:** Gastric bypass, Banded gastric bypass, Restriction, Gastric banding, Bariatric surgery

## Abstract

**Background:**

The gastric bypass is the gold standard of bariatric surgery. Nevertheless some patients show insufficient weight loss or weight regain. Dilation of the pouch or the pouch outlet may be the cause. The banded gastric bypass tries to overcome dilation by placing an implant around the pouch or pouch outlet. In this study we describe our results using the GaBP™ ring system in banded gastric bypass operations in 3 bariatric centers.

**Methods:**

183 patients in 3 bariatric reference centers received a banded gastric bypass operation using the GaBP™ ring system. Up to 4 years follow up was evaluated including weight loss and complications.

**Results:**

Mean EWL after 6 Months was 60% with a mean BMI of 30.1 kg/m^2^. After one year mean EWL reached 75.3% with a mean BMI of 27 kg/m^2^ (110 patients). After two and three years the EWL was 78.8% (n = 49) and 79.9% (n = 35). There was a mean EWL of 85% after 4 years. Thirteen patients finished a 4 year follow up period and mean BMI after 4 years was 25.2 kg/m^2^. In the perioperative and early postoperative period there was a low complication rate (4.3%). Stenosis or dysphagia was observed in only one patient. There was only one ring related complication.

**Conclusion:**

Banded gastric bypass using the GaBP™ ring system allows good weight loss with no regain of weight in a four year follow up. The complication rate is low. A randomized controlled trial is currently underway to compare banded and conventional gastric bypass.

## Background

Gastric bypass surgery was introduced by Edward Mason in 1967. The operation has undergone various modifications. Although there are as many variants of the gastric bypass operation as there are surgeons who perform the operation, the gastric bypass operation is considered the gold standard of bariatric operations [1]. The number of gastric bypass surgeries has increased continuously until 2008. For the first time the number of gastric bypasses performed per year has been decreasing in 2008, mainly to the favor of the gastric banding and the sleeve gastrectomy [2]. This is remarkable because several studies and meta-analyses show that bypass surgery leads to better results than gastric banding, especially in the long term and especially concerning remission of diabetes and cardiovascular disease [3, 4]. A mean excess weight loss of 60% in the first year after the bypass is reported with some weight regain in subsequent years and the side effects and complications are acceptable and treatable [3, 5]. Some authors show that the failure rate increases to 25-40% in patients followed longer than three years because of weight regain. In the meantime studies are published with a follow up of 10 years and failure rates up to 30% [6, 7]. The inadequate weight loss and the weight regain observed in the gastric bypass operation have been attributed mostly to the increase in the gastric reservoir size due to dilatation of the pouch, stoma and proximal small bowel. It is known that either a dilated pouch or a dilated pouch outlet, can lead to a poor restriction, lack of satiety and thus a regain of weight [8–11]. These anatomic landmarks are not routinely evaluated, despite the technical possibility (e.g. pouch volumetry using multi slice CT) [9, 12]. A variation of the gastric bypass has been performed which tries to prevent a dilation of the pouch outlet: the banded gastric bypass. It was described by Capella and Fobi who used the gastric bypass as a revisionary operation after failed VBG, leaving the band in place to prevent complications [13, 14]. Seeing the good results of the bypass with the old VBG-band still in place he performed the primary banded gastric bypass using a silicone band later on. Up to now several banding materials have been used, custom made silicone bands, marlex-meshs and the GaBP™-Ring autolock system [15–17]. We undertook a study to evaluate the weight loss and complication rate after the banded gastric bypass using the GaBP™-Ring (Bariatec Corporation, Palos Verdes Peninsula, CA, USA) system which is a pre-formed ring with a locking mechanism.

## Methods

### Patients

A total of 183 consecutive bariatric patients who agreed for GaBP™ implant were operated with Banded Gastric Bypass between August 2007 and December 2010 in three bariatric reference centers. Among the patients were 118 women and 65 men, mean BMI before the operation was 42.8 kg/m^2^, mean excess weight (calculated as actual weight minus ideal weight based on Broca index (male: height (cm) -100 × 0.9, female: height (cm)-100 × 0.85) was 60.9 kg)). Preoperative patient characteristics are shown in Table 1. Patient weight and BMI were recorded prior to the operation, 3 and 6 months after the operation and 1, 2, 3 and 4 years after the operation. Postoperative complications were also evaluated. Fifteen patients had bariatric surgery before, 13 of them failed gastric banding and 2 sleeve gastrectomy. The study was approved by the Ethics-commission of the University of Freiburg (reference number 321/13). Written informed consent was obtained from all participants.

**Table 1:** **Preoperative patient characteristics**

	Mean	Min	Max	Standard deviation
Weight	121 kg	84 kg	225 kg	19.7 kg
Height	168 cm	148 cm	190 cm	8.5 cm
Body mass index	42.8 kg/m^2^	32 kg/m^2^	72 kg/m^2^	5.9 kg/m^2^
Excess weight	60.9 kg	27 kg	148 kg	16.7 kg
Male : Female	65 : 118			

### Operative technique

**Figure 1:**
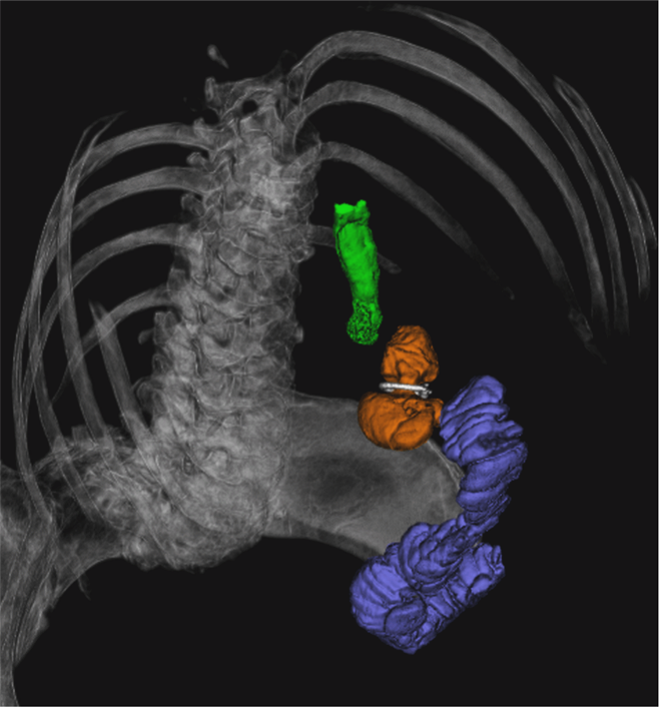
**The banded gastric bypass using the GaBP™-ring.** 3D-reconstruction of abdominal multi slice computed tomography: green: oesophagus, orange: gastric pouch, white: GaBP ring, blue: jejunum.

All the cases were done laparoscopically. A vertical tubular pouch 5–6 cm long is formed using linear staplers. The GaBP™ Ring is placed 4 cm from the angle of His (Figure 1). It is closed according to the manufacturer’s instructions and fixed with two sutures. Rings with a circumference of 6.5 cm (diameter of closed ring is 1.9 cm) were used in all patients. The alimentary limb is created by dividing the jejunum 50 cm below the ligament of Treitz. The gastroenterostomy is performed in an antecolic manner using a circular stapler or hand sewing anastomosis. The integrity of the anastomosis is tested with methylene blue. Detailed operative technique is described elsewhere [18].

### Statistics

Microsoft Excel® (Redmont, WA, USA) and GraphPad Prism® software (San Diego, CA, USA) was used for statistical and graphical analysis.

## Results

**Figure 2:**
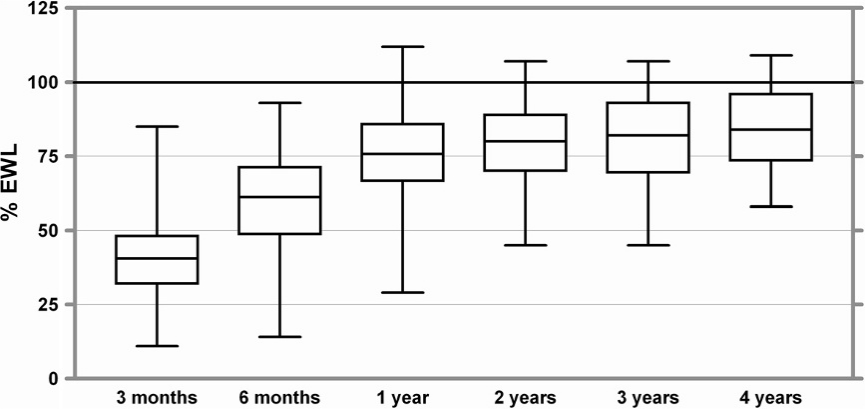
**Excess weight loss up to four years after banded gastric bypass.** Boxes indicate the 25th percentile and the 75th percentile, with a line at the median (the 50th percentile). The whiskers show the highest and lowest values.

**Figure 3:**
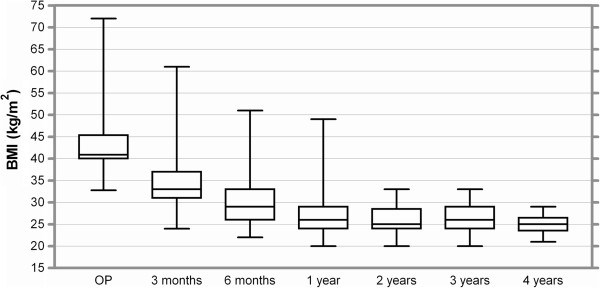
**Reduction of BMI up to four years after banded gastric bypass.** Boxes indicate the 25th percentile and the 75th percentile, with a line at the median (the 50th percentile). The whiskers show the highest and lowest values.

Weight loss: For 147 patients 6 months follow up data was available, Mean EWL after 6 Months was 60% with a mean BMI of 30.1 kg/m^2^. After one year mean EWL reached 75.3% with a mean BMI of 27 kg/m^2^ (n = 110). After two and three years the EWL was 78.8% (n = 49) and 79.9% (n = 35). There was a mean EWL of 85% after 4 years. Thirteen patients finished a 4 year follow up period and mean BMI after 4 years was 25.2 kg/m^2^. Complete date concerning weight loss is displayed in Table 2. Figures 2 and 3 show EWL and reduction of BMI as box plots.

**Table 2:** **Mean excess weight loss (EWL) and mean reduction of body mass index (BMI) up to four years after banded gastric bypass**

Timepoint	n	Mean EWL	SD EWL	Mean BMI (kg/m ^2^)	SD BMI (kg/m ^2^)
Operation		-	-	42.8	5.9
3 months	183	41.0 %	12.1 %	33.9	4.9
6 months	147	60.0 %	15.9 %	30.1	5.1
1 year	110	75.2 %	16.6 %	27.0	4.8
2 years	49	78.8 %	14.7 %	26.0	3.1
3 years	35	79.9 %	15.4 %	26.0	3.1
4 years	13	85.0 %	15.0 %	25.2	2.4

Complications: All operations were done laparoscopically and there was no conversion to open surgery. In the perioperative and early postoperative period there were 8 (4.3%) complications.

– One case of an intraoperative bleeding (0.5%) which made a splenectomy necessary,
– two patients had postoperative intraabdominal bleedings and another two developed intraluminal bleedings which could be treated conservatively (2.1%),
– in two cases (1%) an intestinal perforation was observed which resulted in revisionary surgery, It was not necessary to remove the rings.
– One patient had a cardiac arrest (0.5%) in the early postoperative period with complete recovery.

One female patient had a diagnostic laparoscopy 18 months after the operation due to symptoms of stenosis and abdominal pain, the ring was surprisingly found to be broken in this patient and was replaced. We did not observe any clinical signs for migration or ring slippage in our series; the patients in our follow up did not mention dysphagia or regurgitation as a problem except in one case. Median follow up time for complications was 12 months (3–48 months).

## Discussion

Gastric bypass still is the gold standard in bariatric surgery. However, a relevant subgroup of the operated patients shows insufficient weight loss or even weight regain which might also be accomplished by aggravation or re-occurrence of obesity-associated comorbidities. This subgroup is reported to be between 10 and 30% of the operated patients after 10 years, it might be even bigger in the further follow up [6]. Studies are controversial concerning the question if initial pouch size correlates with weight loss in the further follow up and less is known what happens with the gastric pouch, the pouch outlet, first intestinal loop and lower esophageal sphincter after years in a patient with good and sustainable weight loss [11, 19].

There is also no data concerning the question how long the food stays in the gastric pouch and how fast it is emptying.

On the other hand anatomical reasons can be found in many but not all patients with regain of weight, these are dilation of the gastric pouch and dilation of the gastric pouch outlet which might also lead to a subsequent dilation of the Roux limb resulting in an enlarged functional gastric volume [9]. Dilation of the pouch or pouch outlet as a frequent cause of weight regain was already described by MacArthur in 1980 [20]. In a recently published study by Yimcharoen et al. including 205 patients with weight regain, a dilated gastrojejunostomy was seen in 71%, a dilated pouch in 29% and a combination of both in 12% using a dedicated endoscopical measuring system [8]. In our series of patients with regain of weight or other complications after gastric bypass surgery we noticed pouch dilations in 10 of 18 cases and dilations of the pouch outlet in 8 of 18 cases, often in combination with a dilated Roux limb [9]. This observation leads us to focus our attention to the banded Gastric Bypass technique.

In a study by Mali et al. including patients after silicone ring banded gastric bypass an enlarged gastrojejunostomy could be correlated with a reduced weight loss [21]. The diameter of the outlet (defined as the area inside the pouch where the ring was placed, not the anastomosis) was measured endoscopically one and two years after the operation.

Since implants are commonly used in bariatric surgery it was obvious to use them to prevent pouch outlet dilation. It was done by Capella and by Fobi who placed the ring proximal to the anastomosis [14, 22]. Two similar forms of the banded gastric bypass have been described by Fobi: the siliastic ring Roux-en-Y gastric bypass and later on the Fobi pouch operation which includes the stomach is transsected and the stapler line is covered with the jejunal roux-limb to prevent gastro-gastric fistulas [13]. The evolution from the stapled banded bypass to the transected banded bypass to the transected bypass with interposition of a jejunal loop and the change in complications has also been described by Salinas [23].

Different techniques and different banding materials have been used by others. Bessler and others implanted synthetic meshed materials whereas others use siliastic materials which can be self-made from catheters etc. or industrially manufactured like the GaBP™ ring system [16, 17]. Synthetic meshes have the disadvantage to be incorporated into scar tissue and thus are difficult to remove in case of complications like strictures or stenosis. The same might be the case for fascia lata grafts [24]. Dillemans described the technique of implanting an adjustable gastric band around the pouch in super obese patients [25]. Bessler uses an adjustable band in revisional operations after failed gastric bypass [26]. Recently the placement of banding materials after sleeve gastrectomy has also been published [27–29].

For our study we used the GaBP™-ring system which was also first used by Fobi for gastric bypass surgery. In an initial study of 50 patients in a one year follow up, no ring-related complications were seen with a weight loss comparable to banded gastric bypass using other banding materials [30]. The ring is available in various defined sizes and thus delivers comparable results, especially for a multicenter analysis.

Several studies report short and long term results after banded gastric bypass surgery.

Valezi et al. presented data from 134 patients up to 8 years after banded gastric bypass using 6,5 cm silicone catheters. EWL was 67.6% after one year, 74.3% after two years and slightly decreased again to 69.6% in the fifth and 66% in the eighth year [31]. Salinas et al. report an EWL of 83% 5 years after the operation [32].

In a prospective randomized trial of Bessler et al. comparing banded and non-banded bypass, there was no difference in EWL for the first and second year, but after three years patients in the banded bypass group had a better EWL (73.4% versus 57.7%) [17]. White et al. reported an EWL of 89% one year and 75% ten years after banded bypass using a silicone ring. They also saw a correlation between removal of the ring and regain of weight [33]. Herrera et al. found no difference in weight loss between banded and non banded bypass but they only reported a two year follow up [34]. In a systematic review O’Brien also states that weight loss after banded gastric bypass is better than after short limb or ling limb non banded gastric bypass [35].

In our up to four years follow up we seen no regain of weight. EWL is 75.2% after one year and further increases to 85% EWL after four years, whereas in our collective of standard gastric bypass patients a slight regain in weight can be observed in the third and fourth year after the operation. Thus we assume that ring implantation on the gastric pouch can indeed prevent pouch outlet and first jejunal limb dilation. Since outlet dilation will probably not occur in the first year after gastric bypass, the effect of the banding is likely to be seen in a more than 3 year follow up.

The fact that the banded bypass is not routinely used by most bariatric surgeons might be due to the fear of additional complications like infection, band or ring erosion, migration or stenosis [18]. Band erosion occurs in 1-2% of patients and can mostly be treated by endoscopical removal of the implant [36]. There is no good data concerning the incidence of stenosis after banded bypass, Swain et al. reported 6 cases, where the band could be easily removed laparoscopically [37, 38]. Schwartz et al. published a 3.2% stenosis rate using fascia-lata grafts as a banding, patients were treated with endoscopical dilation which resulted in perforations in 8 out of 32 patients [24]. Dumping syndrome is reported to occur in 24% of non diabetic and even 45% of diabetic patients after banded bypass surgery [39].

Fobi reported a 6% revision rate in a 7 year follow up after transsected siliastic banded bypass. In the study by White et al., ring removal was necessary in 7% of patients due to stenosis, but the need for ring removal might depend on the used implant and on the diameter of the ring [33]. Herrera et al. reported mesh removal in 1 of 30 patients [34]. In our opinion siliastic materials should be used to allow an easy laparoscopic or endoscopic removal in case of complications.

In our patients we saw no ring-related complications besides one case where the ring was broken and had to be replaced. We saw no clinical signs of ring migration; however, we did not do scheduled gastroscopies, so we cannot rule out silent migrations and ring migration might still be a problem in the longer follow up.

We had two patients with intestinal perforations and local peritonitis, but in these cases the rings were not affected and could be left in place.

## Conclusion

To our knowledge this is the first report of up to four years multicenter results using the GaBP™-ring for banded gastric bypass surgery. In the four year follow up we see a good weight loss in the first year and a further slight weight loss up to year four with no regain of weight. However, one limitation of our study is that to date only 13 patients completed the 4 year follow. We assume that banding the pouch can prevent pouch outlet dilation and thus reduce the need for revisionary operations after gastric bypass surgery. The GaBP™-ring is a preformed and auto-locking implant which can be routinely used for the banded bypass and is easily to remove in case of complications. We are looking forward to see the results of multicenter prospective comparison of banded and conventional gastric bypass.
